# Is lymph node dissection mandatory among early stage endometrial cancer patients? A retrospective study

**DOI:** 10.1186/s12905-020-01128-w

**Published:** 2020-11-19

**Authors:** Guangmin Zhang, Hongyou Chen, Yanying Liu, Liyan Niu, Liming Jin, Dong Li, Lihua Song, Lifei Shang, Xiangya Lin, Fei Wang, Fengtong Li, Xinyu Zhang, Xiaoyu Zhang, Yan Gao, Dongyu Qiu, Yunpu Zhang, Ren Na, Riguge Su

**Affiliations:** 1 Chifeng Second Hospital, Chifeng, 024000 Innermongolia China; 2 Inner Mongolia University for the Nationalities, Tongliao, 028043 Innermongolia China

**Keywords:** Endometrial cancer, Lymph node dissection, Low risk, PET-MRI, Metastasis

## Abstract

**Background:**

Whether routine lymph node dissection for early endometrial cancer is beneficial to survival is still controversial. However, surgeons usually perform lymph node dissection on all patients with early endometrial cancer. This study aimed to prove that the risk of lymph node metastasis, as defined by our standard, is very low in such patients and may change the current surgical practice.

**Methods:**

36 consecutive patients who had staged surgery for endometrial cancer were collected. All eligible patients meet the following very low risk criteria for lymph node metastasis, including: (1) preoperative diagnosis of endometrial cancer (preoperative pathological diagnosis), (2) tumors confined to the uterine cavity and not beyond the uterine body, (3) PET-MRI lymph node metastasis test is negative. PET-MRI and pathological examination were used to assess the extent and size of the tumor, the degree of muscular invasion, and lymph node metastasis.

**Results:**

The median age at diagnosis was 52 years (range 35–72 years). The median tumor size on PET-MRI was 2.82 cm (range 0.66–6.37 cm). Six patients underwent robotic surgery, 20 underwent laparoscopic surgery, 8 underwent Laparoscopic-assisted vaginal hysterectomy, and 2 underwent vaginal hysterectomy. 23% (63.9%) patients had high-grade (i.e. 2 and 3) tumors. Among the 36 patients who underwent lymph node sampling, the median number of lymph nodes retrieved was 32 (range 9–57 nodules). No patient (0%) was diagnosed with lymph node metastasis. According to the policy of each institution, 8 patients (22.2%) received adjuvant therapy, and half of them also received chemotherapy (4 patients; 50%).

**Conclusions:**

None of the patients who met the criteria had a pathological assessment of lymph node metastasis. Omitting lymph node dissection may be reasonable for patients who meet our criteria.

## Background

Endometrial cancer is the sixth most common cancer in women worldwide and the most common gynaecological malignancy in developed countries [1]. Wang et al. [2] have reported that about 73% of patients with endometrial cancer are diagnosed in stage I, and the 5-year overall survival rate after surgery is 85% to 91%. The rate of lymph node metastasis in patients with endometrial cancer was reported to be less than 10% [3]. Thus, it is controversial whether all patients with early endometrial cancer need lymph node dissection (LND) [3–5]. LND not only leads to an increase in the incidence of intraoperative complications, but also increases the risk of postoperative lymphocystosis, lower extremity edema, and deep vein thrombosis (DVT) [6, 7]. At the same time, LND will prolong the operation time and hospitalization day, which increase the economic burden of patients.

To prevent unnecessary lymph node dissection, researchers attempt to demonstrate the ophthalmic safety of omitting lymph node dissection in patients with a low risk of lymph node metastasis. Mell et al. [8] analyzed the prognostic data of 58,172 endometrial cancer patients from NCI (National Cancer Institute) from 1988 to 2006. The results showed that for endometrial cancer stage I patients with low-risk, LND group did not improve survival compared with no-LND group. The mortality rate was lower in no-LND group, and the difference was statistically significant (*p* < 0.05). Although many studies suggest that routine lymphadenectomy is not recommended for patients undergoing initial surgery for early endometrial cancer [9]; however, surgeons routinely performed lymph node dissection on all patients, regardless of tumor stage or characteristics [10, 11]. This indicates that the safety evidence of omitting lymph node dissection in low-risk patients is insufficient to prompt surgeons to change their practices.

PET/MRI, as a multimodal molecular imaging technology, integrates positron emission tomography and magnetic resonance imaging, respectively, and has been shown to play an important role in the diagnosis and treatment of endometrial cancer. A retrospective analysis of Kitajima et al. in Japan revealed that the diagnostic accuracy of PET-MRI for lymph node metastasis was 96.7% [12]. Modified with the Mayo Clinic’s criteria for low-risk patients with stage I endometrial cancer, we determined that eligible patients with low risk of lymph node metastasis met the following conditions: (1) preoperative diagnosis of endometrial cancer (preoperative pathological diagnosis), (2) tumors confined to the uterine cavity and not beyond the uterine corpus, (3) PET-MRI examination of lymph node metastasis was negative. The purpose of this study is to demonstrate that the patients defined by our criteria have a very low risk of lymph node metastasis, which could omit lymph node dissection.

## Methods

The cohort being studied was retrospectively recruited from Department of Gynecologic Oncology, Chifeng Second Hospital. The study protocol was revised and accepted by ethics committee of the Chifeng Second Hospital. We included 36 consecutive patients who had staged surgery for endometrial cancer between 2016 and 2018. Bilateral salpingo-oophorectomy and peritoneal irrigation cytology were at the discretion of the surgeon [13]. All eligible patients met the following very low risk criteria for lymph node metastasis, including: (1) Preoperative diagnosis of endometrioid cancer (preoperative pathological diagnosis), (2) tumors confined to the uterine cavity and not beyond the uterine corpus, (3) PET-MRI examination of lymph node metastasis was negative. The Chifeng Second Hospital granted Ethical approval to carry out the study within its facilities (Ethical Application Ref: CF-2016058).

All patients received an integrated PET/MRI scanner BiographmMR (Siemens Healthcare Department in Erlangen, Germany) before the operation, and the results were interpreted by the radiologist. We used PET-MRI to assess the extent and size of the tumor, the degree of muscular invasion, and lymph node metastasis. Exclude any patients with suspected lymphadenopathy.

The surgical specimens were evaluated by pathologists who specialize in gynecological pathology, and were blinded to the patient's results. We obtained the final pathological results, including the stage according to the International Federation of Obstetricians and Gynecologists (FIGO), the type and grade of histology, the presence of malignant cells in peritoneal flushing cytology, the number of recovered lymph nodes, and the involved lymph nodes. The final pathological results indicate that the patients are middle-risk or high-risk, and receive adjuvant treatment based on the postoperative results [13].

## Results

### Preoperative patient characteristics and operation-related details

**Fig. 1:**
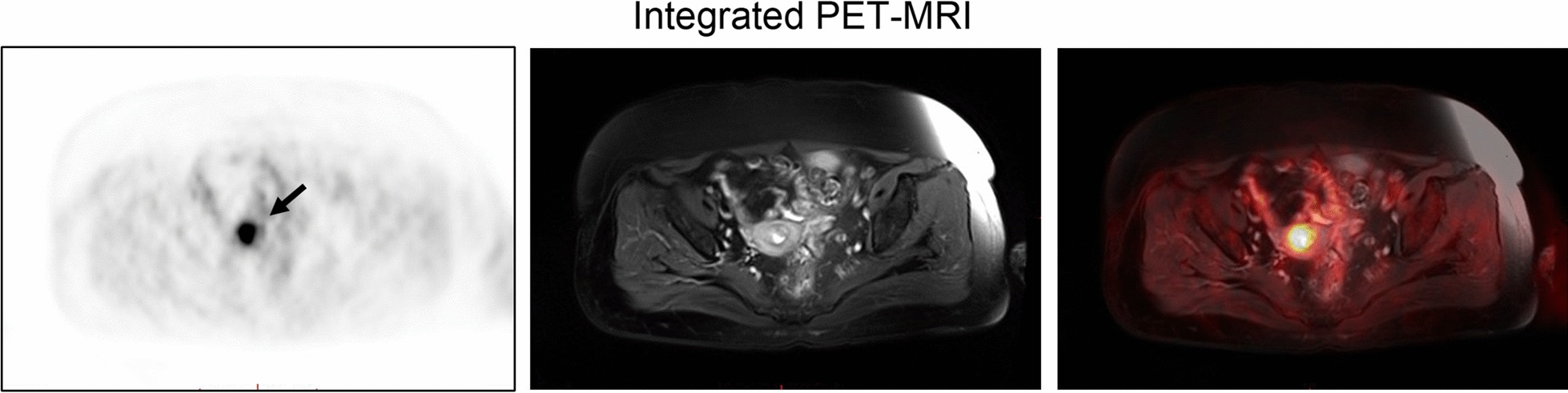
One patient with endometrial cancer. Axial integrated PET/MRI shows intense ^18^F-FDG uptake by uterine cavity (black arrow).

Table 1 summarizes the preoperative characteristics of the study population. Before surgery, based on endometrial biopsy, all 36 patients were diagnosed with endometrial histology (Table 1). The median age at diagnosis was 52 years (range 35–72 years). The median tumor size on PET-MRI was 2.82 cm (range 0.66–6.37 cm) (Fig. 1). The average value of standard uptake value (SUV) and apparent diffusion coefficient (ADC) on PET-MRI were 5.62 and 821.2 respectively (range 1.44–11.03 and 477.00–11,125.0, respectively). All patients underwent initial staging including hysterectomy (Table 2). Six patients underwent robotic surgery, 20 underwent laparoscopic surgery, 8 underwent Laparoscopic-assisted vaginal hysterectomy, and 2 underwent vaginal hysterectomy. At the discretion of the surgeon, bilateral salpingo-oophorectomy (34 cases; 94.4%) and peritoneal lavage cytology (30 cases; 83.3%) were selected. The surgeon performed pelvic lymph node sampling and para-aortic lymph node sampling in all 36 patients (100%).

**Table 1:** Preoperative patient characteristics (n = 36)

Parameters	Value
Age (year)	52 (35–72)
Body mass index (kg/m^2^)	25.5 (18.5–36.0)
Tumor size on PET-MRI (cm)	2.82 (0.66–6.37)
SUV average on PET-MRI	5.62 (1.44–11.03)
ADC average on PET-MRI	821.2 (477.0–1125.0)

The values are presented as the median (range) or number (%), unless otherwise indicated

**Table 2:** Surgical details (n = 36)

Parameters	n (%)
Approach	
Robotic system (da Vinci surgical technique)	6 (16.6)
Laparoscopic	20 (55.6)
Laparoscopic-assisted vaginal	8 (22.2)
Vaginal	2 (5.6)
Bilateral salpingo-oophorectomy	
Yes	34 (94.4)
No	2 (5.6)
Peritoneal washing cytology	
Yes	30 (83.3)
No	6 (16.7)
Pelvic lymph node sampling	
Yes	36 (100)
No	0 (0)
Para-aortic lymph node sampling †	
Yes	36 (100)
No	0 (0)

### Final pathologic results and clinical course

The results of the final pathological examination after surgery are shown in Table 3. The tumors of 34 patients were limited to < 50% (94.4%) of the myometrium. Contrary to the preoperative expectation, the tumor extended to ≥ 50% the myometrium in 1 patient (2.8%) and beyond the uterine corpus in 1 patient (2.8%). No patient was diagnosed with FIGO stage III cancer with lymph node metastasis. In all patients, the histological type was confirmed to be endometrioid. Twenty-three (63.9%) of patients had high grade (i.e. 2 and 3) tumors. Of the 30 patients evaluated by peritoneal irrigation cytology, 2 (6.7%) had malignant cells in the irrigation fluid. Among the 36 patients who underwent lymph node sampling, the median number of lymph nodes retrieved was 32 (range 9–57 nodules). No patient (0%) was diagnosed with lymph node metastasis. According to the policy of each institution, 8 patients (22.2%) received adjuvant therapy, and half of them also received chemotherapy (4 patients; 50%).

**Table 3:** Final pathology details and clinical course

Parameters	Value
FIGO stage	
IA	34 (94.4)
IB	1 (2.8)
II	1 (2.8)
III–IV	0 (0)
Histology in postoperative uterine biopsy	
Endometrioid	36 (100)
Nonendometrioid	0
Grade, based on the postoperative uterine biopsy	
1	12 (33.3)
2	18 (50)
3	5 (13.9)
Unknown	1 (2.8)
Malignant cells in peritoneal washing cytology	
Presence	2 (6.7)
Absence	28 (93.3)
Retrieved lymph nodes	32 (9–57)
Lymph node metastasis	
Yes	0 (0)
No	36 (100)
Adjuvant therapy	
Yes	8 (22.2)
Radiotherapy	3 (37.5)
Chemotherapy	4 (50)
CCRT	1 (12.5)
No	28 (77.8)

## Discussion

This study showed that the low risk criteria for lymph node metastasis can predict the probability of lymphatic metastasis in patients with endometrial cancer. None of the patients who met the criteria had lymph node metastasis confirmed by pathology. In this study, 8 (22.2%) patients underwent postoperative adjuvant therapy because of unexpected high-risk pathology, such as > 1/2 myometrial invasion or tumor size > 2 cm. This is also acceptable because we did not rule out these two high-risk factors from the low risk criteria.

Several studies have previously recommended evaluation criteria for endometrial cancer patients with low-risk lymph node metastases and to verify tumor safety in patients who meet the criteria. Mariani et al. [14] used the Mayo criteria to select 328 patients with low risk of lymph node metastasis and found the 5-year recurrence-free survival (RFS) and overall survival (OS) was 96% and 97%, respectively. The criteria are as follows: endometrioid carcinoma, grade 1 or grade 2 tumor, < 50% myometrial infiltration, there is no visible evidence during the operation, and the largest visible area is not greater than 2 cm. However, these criteria incorporate the pathological grade of the tumor, which was obtained after surgery by frozen sections. Bell and Mitamura et al. [15] modified the Mayo criteria and included only patients who did not undergo lymph node dissection. They found the 5-year OS to be 95.8%. Unfortunately, they need to be based on pathological results. Kim et al. [13] recently proposed a new KGOG standard, and the 3-year RFS and 5-year OS were 98.6% and 98.6%, respectively. However, the clinical imaging diagnosis of myometrial invasion and lymph node size had a certain misdiagnosis rate, especially CT.

Our study innovatively incorporates PET-MRI into the low-risk criteria. PET-MRI integrates positron emission tomography and magnetic resonance imaging. Several studies have demonstrated the role of PET-MRI in the diagnosis of endometrial cancer [16, 17], but it is the first time that PET-MRI is included in the low-risk criteria of lymph node metastasis in endometrial cancer. Kitajima et al. [12] found that the sensitivity, specificity and accuracy of PET/MRI for pelvic lymph node metastasis of endometrial cancer were 100%, 96% and 97%. Our study found that none of the patients who met the criteria was evaluated for lymph node metastasis, which proved the efficacy of PET-MRI in predicting lymph node metastasis in endometrial cancer patients.

There are several limitations in our study. First, this study is a retrospective analysis, and the sample size is not large enough. Second, the study did not follow-up analysis of patients' 3- or 5-year survival rates. Third, all the patients had endometroid type on histology. Data on other pathological types of endometrial cancer need to be studied later We are currently collecting and tracking these data and will publish these results in the near future. In addition, these criteria require PET-MRI assistance and may not be applicable in some developing countries.

## Conclusions

Lymph node dissection could be omitted in the endometrial cancer patients who met our criteria. We hope that our findings will change the treatment of endometrial cancer in the future. Multi-center prospective research is also imperative.

## Data Availability

The datasets used and analyze d in the current study are available from the corresponding author upon reasonable request.

## Abbreviations

LND: Lymph node dissection
DVT: Deep vein thrombosis
FIGO: Federation of obstetrics and gynecology
RFS: Recurrence-free survival
OS: Overall survival
